# MODMS: a multi-omics database for facilitating biological studies on alfalfa (*Medicago sativa* L.)

**DOI:** 10.1093/hr/uhad245

**Published:** 2023-11-27

**Authors:** Longfa Fang, Tao Liu, Mingyu Li, XueMing Dong, Yuling Han, Congzhuo Xu, Siqi Li, Jia Zhang, Xiaojuan He, Qiang Zhou, Dong Luo, Zhipeng Liu

**Affiliations:** State Key Laboratory of Herbage Improvement and Grassland Agro-ecosystems, College of Pastoral Agriculture Science and Technology, Lanzhou University, Lanzhou 730020, China; State Key Laboratory of Herbage Improvement and Grassland Agro-ecosystems, College of Pastoral Agriculture Science and Technology, Lanzhou University, Lanzhou 730020, China; State Key Laboratory of Herbage Improvement and Grassland Agro-ecosystems, College of Pastoral Agriculture Science and Technology, Lanzhou University, Lanzhou 730020, China; State Key Laboratory of Herbage Improvement and Grassland Agro-ecosystems, College of Pastoral Agriculture Science and Technology, Lanzhou University, Lanzhou 730020, China; Tropical Crops Genetic Resources Institute, Chinese Academy of Tropical Agricultural Sciences, Haikou 571101, China; State Key Laboratory of Herbage Improvement and Grassland Agro-ecosystems, College of Pastoral Agriculture Science and Technology, Lanzhou University, Lanzhou 730020, China; State Key Laboratory of Herbage Improvement and Grassland Agro-ecosystems, College of Pastoral Agriculture Science and Technology, Lanzhou University, Lanzhou 730020, China; State Key Laboratory of Herbage Improvement and Grassland Agro-ecosystems, College of Pastoral Agriculture Science and Technology, Lanzhou University, Lanzhou 730020, China; State Key Laboratory of Herbage Improvement and Grassland Agro-ecosystems, College of Pastoral Agriculture Science and Technology, Lanzhou University, Lanzhou 730020, China; State Key Laboratory of Herbage Improvement and Grassland Agro-ecosystems, College of Pastoral Agriculture Science and Technology, Lanzhou University, Lanzhou 730020, China; State Key Laboratory of Herbage Improvement and Grassland Agro-ecosystems, College of Pastoral Agriculture Science and Technology, Lanzhou University, Lanzhou 730020, China; State Key Laboratory of Herbage Improvement and Grassland Agro-ecosystems, College of Pastoral Agriculture Science and Technology, Lanzhou University, Lanzhou 730020, China

## Abstract

Alfalfa (*Medicago sativa* L.) is a globally important forage crop. It also serves as a vegetable and medicinal herb because of its excellent nutritional quality and significant economic value. Multi-omics data on alfalfa continue to accumulate owing to recent advances in high-throughput techniques, and integrating this information holds great potential for expediting genetic research and facilitating advances in alfalfa agronomic traits. Therefore, we developed a comprehensive database named MODMS (multi-omics database of *M. sativa*) that incorporates multiple reference genomes, annotations, comparative genomics, transcriptomes, high-quality genomic variants, proteomics, and metabolomics. This report describes our continuously evolving database, which provides researchers with several convenient tools and extensive omics data resources, facilitating the expansion of alfalfa research. Further details regarding the MODMS database are available at https://modms.lzu.edu.cn/.

## Introduction

Alfalfa, widely recognized as the ‘king of forages’, is known for its exceptionally high protein content, adaptability to various environments, ease of cultivation, and rapid growth [1]. Owing to the burgeoning demand for meat, eggs, milk, and other livestock products, it is widely recognized as one of the most economically valuable crops worldwide [2, 3]. Alfalfa is grown extensively in the arid and semi-arid northern regions of China, where diverse environmental factors often constrain its yield and quality [4]. Therefore, uncovering the mechanisms underlying the crucial agronomic traits and stress responses in alfalfa is of great importance.

Recently, multi-omics technologies, such as genome-wide association studies, transcriptomics, proteomics, and metabolomics, have emerged as powerful tools for exploring the mechanisms underlying critical agronomic traits and stress responses in alfalfa [5–8]. Over the past 3 years, four high-quality genome assemblies of cultivated alfalfa species have been released, namely Zhongmu No.1, Zhongmu No.4, XingjiangDaye, and *Medicago sativa ssp. caerulea*[9–12]. Numerous transcriptomic datasets have been published, including four related to biotic stress [13–16], five related to abiotic stress [8, 17–20], and seven related to important agronomic traits [21–26]. These studies have identified genes involved in metabolic pathways, such as the abscisic acid response, glutathione and sulfur metabolism, and secondary metabolic pathways, which are crucial in responding to various external stresses [8, 18]. These studies have also revealed the critical role of autumn dormancy in the winter adaptability of alfalfa [23, 24]. Furthermore, a small but growing body of proteomics and metabolomics research has shed light on the potential mechanisms underlying critical agronomic traits and stress responses in alfalfa [7, 27, 28]. Notably, genomic variants, including single nucleotide polymorphisms (SNPs), insertions and deletions (InDels), and structural variations (SVs), have garnered attention owing to their significant roles in plant adaptive evolution and diversification. However, a comprehensive survey of these types of genetic variations in alfalfa, including their distributions and frequencies within populations, is still lacking.

The availability of datasets of these types could significantly contribute to enhancing the understanding of stress responses, plant growth and development, genetic diversity, domestication, and origins. Many databases have been constructed to store and leverage the wealth of datasets of these types, such as TVIR (http://tvir.bio2db.com), a comprehensive vegetable information resource database for comparative and functional genomic studies [29]; the Hsf database (http://hsfdb.bio2db.com), a database of heat shock transcription factors [30]; CitGVD, (http://citgvd.cric.cn/), a comprehensive database of citrus genomic variations [31]; CottonMD (https://yanglab.hzau.edu.cn/CottonMD), a comprehensive database that integrates genomic, metabolic, and transcriptomic data from 508 maize individuals, enabling various types of quantitative trait locus (QTL) analyses, including phenotypic QTLs, expression QTLs, and metabolic QTLs [32]; and GRAND (http://grand.cricaas.com.cn), containing genomic and transcriptomic information from *Gossypium* species [33]. Similarly, several databases related to *Medicago* species have also been developed, such as AGED (http://plantgrn.noble.org/AGED/), a web-accessible gene expression atlas for investigating expression differences between *M. sativa* subspecies; alfaNET (http://bioinfo.usu.edu/alfanet/), a database of protein–protein interactions between alfalfa and bacteria, providing genomic, gene expression, and pan-genomic resources for alfalfa research and breeding [26, 34]; MtExpress (https://lipm-browsers.toulouse.inra.fr/pub/expressionAtlas/app/mtgeav3/), a comprehensive and curated RNA-seq-based gene expression atlas for the model legume, *Medicago truncatula* [35]; Symbimics (https://iant.toulouse.inra.fr/symbimics/), comprising the laser dissection and transcriptomic resources of *M. truncatula* [36]; LeGOO (https://lipm-browsers.toulouse.inra.fr/k/legoo/), an expertized knowledge database for the model legume, *M. truncatula* [37]; and MtrunA17r5.0-ANR (https://medicago.toulouse.inra.fr/MtrunA17r5.0-ANR/), the genome portal of *M. truncatula* A17 [38]. However, despite their positive reference values and roles in their respective fields, these databases are mostly constructed based on *M. truncatula*, with less emphasis on alfalfa and primarily consist of single-omics datasets.

In this study, we have developed a comprehensive database, called MODMS. The goal of this is to enhance functional genomics research and accelerate researcher efforts to improve the productivity and sustainability of alfalfa production. We further provide a detailed description of the creation and functions of the user-friendly MODMS database.

## Database content and usage

### Structural overview of the MODMS database

**Figure 1:**
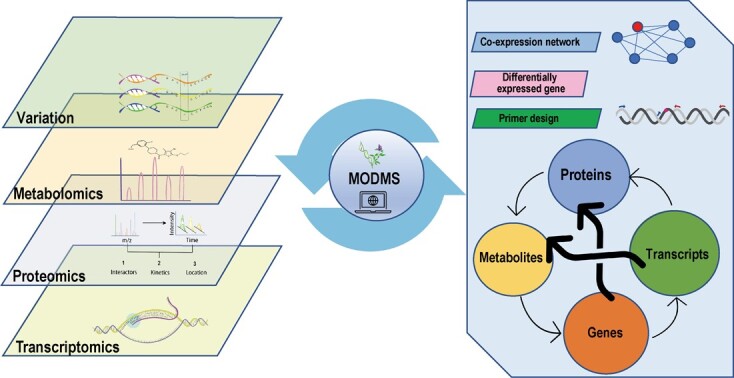
Overview of MODMS, including the construction pipeline for multi-omics data integration.

**Figure 2:**
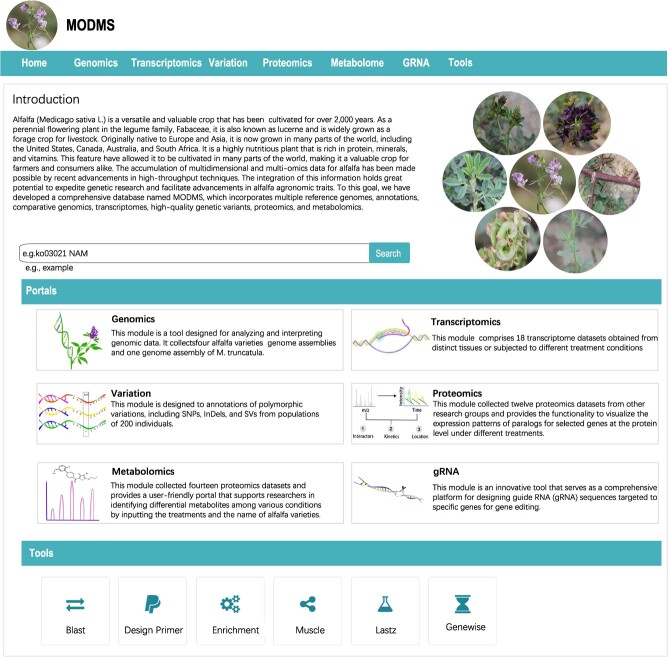
Screenshot of the MODMS home page. The MODMS home page features a top menu bar, a site-wide search engine, access to six biological modules, and a range of bioinformatics tools.

The MODMS database is a user-friendly database for alfalfa, which stores and displays multi-omics datasets to enhance functional genomics research on alfalfa (Fig. 1). It is designed to comprise the following seven components: genomics, transcriptomics, variations, proteomics, metabolomics, guide RNA (gRNA), and tools (Fig. 2). These portals provide abundant and convenient visual tools to browse and compare genome sequences, gene structures, and metabolite contents, and also understand gene regulation and evolutionary mechanisms. The database interface can be accessed directly by entering the URL https://modms.lzu.edu.cn/ on the web page.

### The genomics module

**Figure 3:**
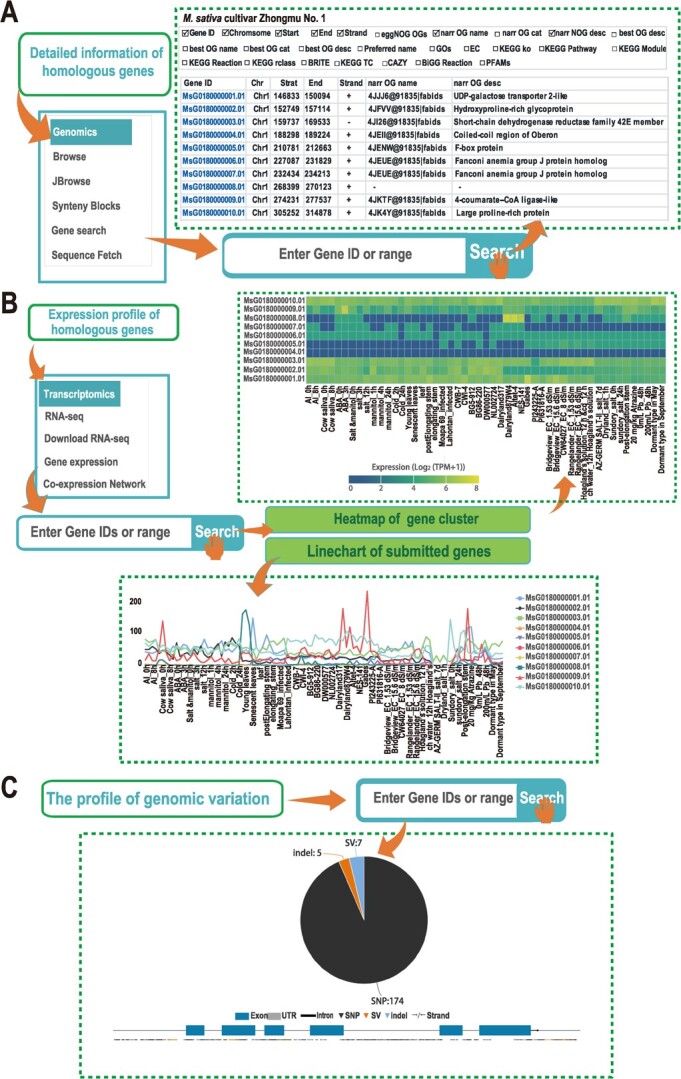
Genomics, transcriptomic, and variation portals in MODMS: (**A**) gene information catalogs, (**B**) expression patterns, and (**C**) descriptions of genomic variation. The expression values are shown in the binary logarithm of transcripts per million (TPM) + 1.

The genomics module is a tool designed to analyse and interpret genomic datasets. It collects genome assemblies of four alfalfa varieties and *M. truncatula* (Fig. 3A). The primary features incorporated into this module are gene search, sequence fetch, synteny blocks, JBrowse, Browse, and download genome. Gene search and sequence fetch allow users to query targeted genes by inputting gene IDs or their chromosomal locations. The functional annotation of target genes, including Gene Ontology (GO) and Kyoto Encyclopedia of Genes and Genomes (KEGG) pathways. Sequences (CDSs), proteins, and promoter sequences are also obtainable. The genome browser JBrowse is a gene sequence browser embedded in the database designed to facilitate the exploration of the reference sequence of alfalfa, and it provides a graphical display of genome [39]. Each genome assembly has a dedicated page containing general information about the assembly and submenus that link to related information. The Browse feature provides position information and various functional annotations for all genes from different genomes in a list format, without graphical representation. The synteny blocks provide a collinearity analysis between any two of the four alfalfa genome datasets and one genome dataset of *M. truncatula* in the MODMS database. Users can click on the corresponding block ID in the overview interface to view the detailed analysis and obtain a visual collinearity map of these genes. Furthermore, the database emphasizes helitron, long terminal repeat (LTR), and terminal inverted repeat (TIR) sequences in the annotation information. The download genome feature provides researchers access to the genome sequence files of the four alfalfa varieties and *M. truncatula*, which are made available through the database. The genome, CDS, and protein sequence files are provided in FASTA format, whereas the annotation file is available in GFF3 format.

### The transcriptomic module

The transcriptomic module comprises 18 transcriptome datasets obtained from distinct tissues or under different treatment conditions (Table S1, see online supplementary material). This module facilitates the visualization of the expression patterns of selected genes in diverse tissues or under different treatment conditions using a heatmap or line chart, after querying gene IDs or chromosomal locations (Fig. 3B). This resource provides valuable insights into the expression patterns of target genes across multiple transcriptome datasets, enabling researchers to comprehend the transcriptomic changes associated with these treatments and better understand how gene expression could be regulated under specific environmental conditions. Moreover, this module offers comprehensive information on co-expression networks and produces a map displaying the genes exhibiting the highest degree of association (Fig. 3B). This allows researchers to investigate gene–gene associations and provide valuable insights into the complex biological processes involved in gene regulation and functions.

### The variations module

The variations module was designed to collect the annotations of genomic variations, including SNPs, InDels, and SVs, from populations generated by our lab using Zhongmu No.1 as the reference genome. This tool offers valuable information on SNPs, InDels, and SVs across different regions of chromosomes in genomes, enabling researchers to study the genetic differences among different individuals. Users can search for SNPs/InDels/SVs using geneIDs or their locations, and this tool displays the acquired SNPs/InDels/SVs in the gene structures, in the upstream and downstream regions as well as their locations, and their allele frequencies were represented by the size of the triangles (Fig. 3C).

### The proteomics module

**Figure 4:**
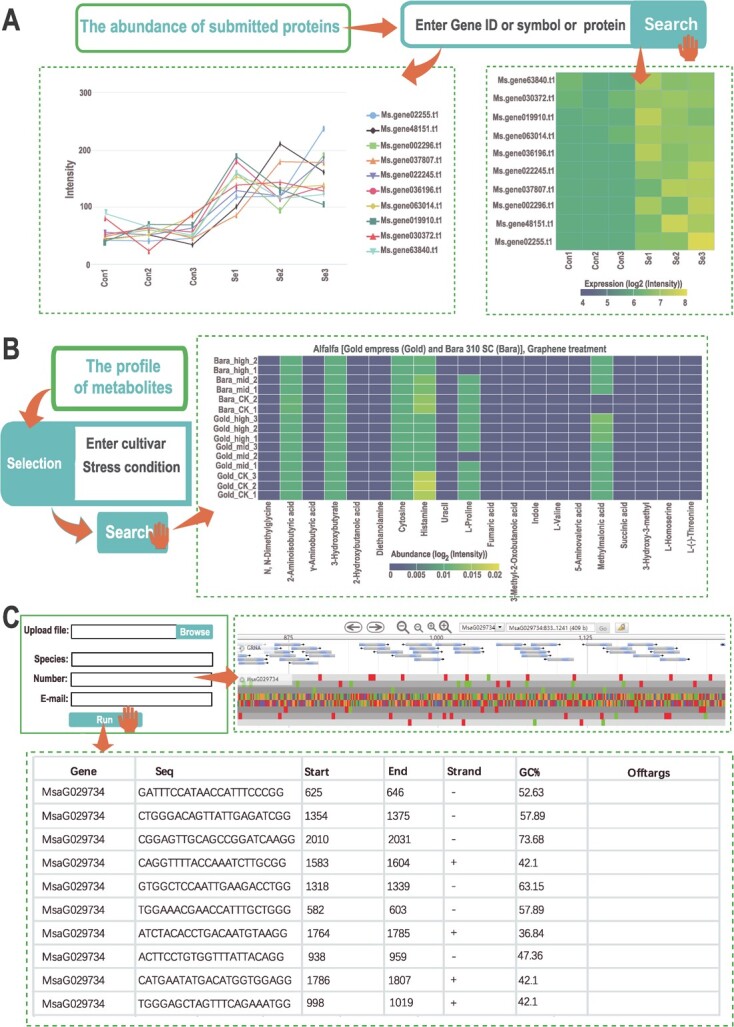
Proteomics, metabolomics, and guide RNA (gRNA) portals in MODMS: (**A**) expression profiles at the proteomic level, (**B**) metabolite profiles in different varieties and under different treatment conditions, and (**C**) small gRNA design for gene editing.

The proteomics module offers access to 12 proteomic datasets from other research groups (Table S2, see online supplementary material). The portal provides the functionality to visualize the expression patterns of the paralogs of selected genes at the protein level under different treatment conditions. Heatmaps can also be generated by querying the gene IDs of the proteins of interest and providing a list of protein expression values under distinct conditions (Fig. 4A).

### The metabolomics module

The metabolomics module, a collection of 13 metabolomics datasets, provides a user-friendly portal that supports researchers in identifying differential metabolites among various conditions by inputting the treatments and the names of alfalfa varieties (Table S3, see online supplementary material). The module also supports the visualization of metabolite differences among various conditions based on a heatmap (Fig. 4B).

### The gRNA module

The gRNA module is an innovative tool that serves as a comprehensive platform for designing gRNA sequences targeting specific genes for gene editing. This platform offers a comprehensive solution for the rapid and efficient design of gRNA sequences that can be utilized in CRISPR-Cas9 systems to induce precise and targeted changes in the DNA sequences. By inputting a gene sequence of interest and an email address, researchers can design gRNA sequences that precisely target specific locations within the target gene (Fig. 4C). It also provides information on potentialoff-target effects and recommends alternative gRNA sequences to reduce these effects (Fig. 4C).

### The tools module

The tools module includes eight analytical processing tools, namely, BLAST, Design Primer, Enrichment Analysis, Muscle, LASTZ, Genewise, SNP Heatmap, and Omics Network Analysis. These tools are available to users for their research in combination with MODMS to obtain the desired results. The BLAST server [40] is an integrated information alignment tool that is similar to the NCBI BLAST tool but is simpler and more user-friendly. The BLAST gene tool is commonly used to align genes using CDSs, and the BLASTN genome uses a heuristic algorithm to identify regions of similarity between the genomes of two species. In contrast, BLASTP is the preferred tool for aligning genes based on protein sequences. Users can either input sequences directly or upload files and modify relevant parameters, such as the E-value, word size, and maximum target sequences during the query. The Designed Primer tool offers primer design services for specific chromosomal locations and target sequences known to users. The Enrichment Analysis tool is often used in conjunction with other databases, such as the KEGG pathway database or GO database. Additionally, tables and maps are provided for user convenience. Muscle is a tool that offers multi-sequence alignment at the protein level [41]. Users can quickly obtain tree results data and linear tree plots by importing a file in FASTA format. LASTZ is a DNA sequence alignment tool that is specifically designed to align large and complex genomes [42]. It provides various parameters that users can adjust to optimize the alignment results, such as gap penalties and score thresholds. It also outputs alignment results in several formats, including SAM and minor allele frequencies (MAFs) that can be used for downstream analyses. Genewise uses a heuristic algorithm that compares a protein sequence to a genomic DNA sequence and generates an alignment of the two sequences [43]. This alignment can then be used to predict gene structures, including the exon and intron boundaries. The SNP Heatmap can be used to display the frequencies of SNPs. The Omics Network Analysis tool is designed for integrated analysis among multi-dimensional datasets.

### Using the MODMS database: a case study

**Figure 5:**
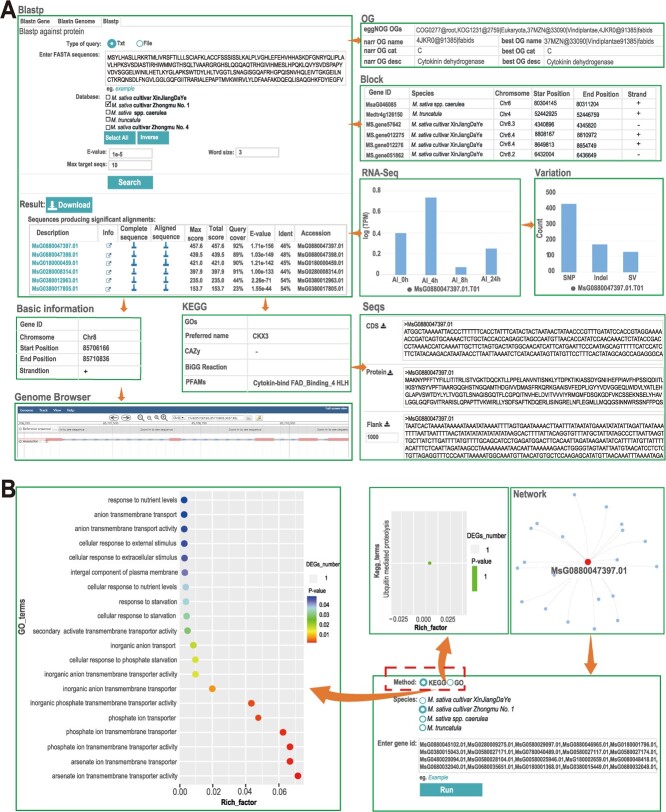
Case study of the cytokinin oxidative dehydrogenase 6 (*CKX6*) gene based on multiple omics data using MODMS. (**A**) Basic information for *MsG0880047397.01* obtained using the MODMS search function, including the gene location (start and end sites and chromosome chains), gene structure, annotation (eggNOG, KEGG, and GO), orthologous genes in the synteny blocks, expression patterns, genomic variation, co-expression network, and the coding, protein, and its flank sequences. (**B**) The GO and KEGG enrichment analysis of the genes co-expressed with *MsG0880047397.01*.

Cytokinin is a crucial plant endogenous hormone that regulates various physiological processes and development, including root morphogenesis and nodule formation and development [44, 45]. Maintaining this dynamic balance is vital for the normal development of plants. Cytokinin oxidative dehydrogenase 6 (CKX6) is the primary enzyme responsible for the irreversible degradation of cytokinins in plants [46]. Thus, we chose *CKX6* as the starting point to test the various functions of MODMS. In the ‘Tools’ module, the ‘BLAST’ interface page was accessed, and the CKX6 protein obtained from the TAIR website (https://www.arabidopsis.org/index.jsp) was submitted for a homologous BLASTP search. The analysis identified six homologous genes from Zhongmu No.1. Next, to obtain the detailed information regarding the best-matched gene ID, in this case, ‘*MsG0880047397.01’*, we clicked the info icon (Fig. 5A), and the ‘Basic Information’ section displayed this information in the genome browser. Moreover, users can find the Evolutionary Genealogy of Genes: Non-supervised Orthologous Groups (eggNOG) annotation, KEGG terms of this gene in these annotation columns. The ‘Block’ column indicated the presence of one, one, and four homologous genes in the *M. truncatula*, *M. sativa* spp*. Caerulea*, and *M. sativa* cultivar XinJiangDaYe genomes, respectively. This was visualized using a Circos plot (Fig. S1, see online supplementary material). The ‘RNA-Seq’ column provided information on the expression pattern of *MsG0880047397.01* across different conditions or developmental stages. The ‘Variation’ column provided information on polymorphic variations identified in this gene, including SNPs, InDels, and SVs. The ‘seqs’ column provided sequences of the CDS, protein, and gene upstream regions that can be used for promoter analysis or functional studies. Furthermore, a co-expression network for *MsG0880047397.01* was generated, displaying those genes with the highest correlation with this gene (Fig. 5A). Enrichment analysis of these co-expressed genes using online tools revealed that they were enriched in the ubiquitin mediated proteolysis pathway (KEGG analysis), as well as in phosphate ion transport, arsenate ion transmembrane transporter activity, and phosphate ion transmembrane transporter activity (GO analysis) (Fig. 5B).

## Summary and future directions

Alfalfa is a forage crop that has gained attention owing to its excellent characteristics in relation to animal products. However, existing alfalfa databases are limited to basic research fields, such as genome and SNP annotations. To fill this gap, we developed the MODMS database, which includes genomics, transcriptomic, genetic variation, proteomic, and metabolomics datasets of alfalfa. MODMS has several advantages over other previously published databases related to *Medicago* species in the following aspects. It is the first database to offer a comprehensive genetic variation analysis, which is crucial for discovering potential candidate variants or genes. In addition, MODMS provides multiple common bioinformatic analyses and user-friendly search tools that facilitate the rapid acquisition of relevant omics information, enabling the analysis of different types of datasets, including genomics, transcriptomics, proteomics, and metabolomics. Finally, it provides gRNA tools to assist researchers in designing single gRNA sequences that specifically target genes for gene editing purposes, which are particularly valuable in the gene editing field for the precise and targeted manipulation of genes, to elucidate gene functions in alfalfa. Overall, information derived from MODMS can facilitate functional validation and guide the development of breeding strategies aimed at improving crop or livestock productivity.

In the future, continued technological developments and advances will enable the generation of even more extensive and diverse datasets. Our goal with MODMS is to provide an expanded database by incorporating omics data from additional accessions and tissues and using more powerful statistical methods to enhance the accuracy and reliability of the analyses.

## Construction and methods

### Database implementation

The MODMS database was deployed and operated on the Ubuntu 20.04 operating system, utilizing the MySQL database management system for data storage and management. Slick 3.3.2 was utilized as a middleware layer to optimize query performance and ensure efficient data retrieval from the MODMS database. Moreover, JBrowser 1.16.6 (https://www.jbrowse.org), a robust genome browser tool that offers a user-friendly and interactive strategy for visualizing genomic data, was employed to integrate genome visualization functionality into the website. The design and implementation of the website interface were bolstered using Bootstrap 4.6.0 (https://getbootstrap.com) and the Play Framework 2.8.7 (https://www.playframework.com/). This website underwent rigorous testing on prominent web browsers, such as Firefox, Google Chrome, and Internet Explorer, and it demonstrated stable performance.

### Genome sequence and annotation

The MODMS database integrates four alfalfa genome assemblies and one genome assembly of *M. truncatula*, along with their corresponding gene annotations data sourced from pertinent databases or articles [9, 11, 12, 47]. The predicted genes from each of these species were individually annotated against the eggNOG and KEGG databases using eggNOG-mapper 5.0 (http://eggnog5.embl.de) and KOBAS 3.0 (http://kobas.cbi.pku.edu.cn/) at the genome-wide level to retrieve GO terms and KEGG Orthology data, respectively [48, 49].

### RNA-Seq analyses

The MODMS database also collects raw transcriptome datasets from the National Center for Biotechnology Information (NCBI) database for alfalfa based on various treatment conditions, developmental stages, or tissue types (Table S1, see online supplementary material). These datasets were filtered using FastQC (version 0.11.2) to remove low-quality reads. The resulting clean RNA-seq reads were then aligned to the reference genome (Zhongmu No.1) using HISAT2 (version 2.1.0), and the transcript per million (TPM) values were estimated using featureCounts (version 2.0.2) [50]. Subsequently, line charts and heatmaps were generated for each gene using these TPM values.

### Co-expression network construction

The MODMS database can also be used to implement a co-expression network analysis. A co-expression network was constructed using the WGCNA (version 1.70–3) package [51]. This approach uses a weighted correlation matrix to measure the correlation between gene expression patterns across different samples, generating modules based on their co-expression patterns. The resulting co-expression network was visualized using Cytoscape (version 3.6.1), a software tool for visualizing and analysing complex networks [52].

### Synteny block analyses

Synteny blocks and homologous genes can provide insights into the evolutionary history and functional conservation of species. Homologous genes between the reference genome and query genomes were identified using BLASTP (version 2.2.28) with an e-value threshold of 1e^-5^ [53], and MCScanX (version 1.1.11) was used with default parameters to determine the synteny block [54].

### Genomic variation detection

Genome resequencing data from 200 individuals were generated in our laboratory (unpublished). After filtering out low-quality reads and adapters, the clean reads were mapped to the Zhongmu No.1 reference genome using BWA (version 0.7.10-r789) and sorted using SAMtools (version 0.1.19) [55]. The Genomic Analysis Toolkit was then used to filter out genomic variations, such as SNPs, InDels, and SVs, with low mapping quality using the following parameters: QUAL <30.0 || MQ < 50.0 || QD < 2 [56]. Finally, all SNPs and InDels with MAFs <0.01 or missing rates >0.1 were discarded using VCFtools (v.0.1.16) [57].

## Supplementary Material

Web_Material_uhad243
